# Spatiotemporal modeling of first and second wave outbreak dynamics of COVID-19 in Germany

**DOI:** 10.1007/s10237-021-01520-x

**Published:** 2021-10-06

**Authors:** Dorothee Lippold, Andreas Kergaßner, Christian Burkhardt, Matthias Kergaßner, Jonas Loos, Sarah Nistler, Paul Steinmann, Dominik Budday, Silvia Budday

**Affiliations:** 1 Department of Mechanical Engineering, Institute of Applied Mechanics, Friedrich-Alexander-University Erlangen Nürnberg, 91058 Erlangen, Germany; 2 Department of Computer Science, Hardware-Software-Co-Design, Friedrich-Alexander-University Erlangen-Nürnberg, 91058 Erlangen, Germany

**Keywords:** COVID-19, SIQRD model, Mesoscale outbreak dynamics, Germany

## Abstract

The COVID-19 pandemic has kept the world in suspense for the past year. In most federal countries such as Germany, locally varying conditions demand for state- or county-level decisions to adapt to the disease dynamics. However, this requires a deep understanding of the mesoscale outbreak dynamics between microscale agent models and macroscale global models. Here, we use a reparameterized SIQRD network model that accounts for local political decisions to predict the spatiotemporal evolution of the pandemic in Germany at county resolution. Our optimized model reproduces state-wise cumulative infections and deaths as reported by the Robert Koch Institute and predicts the development for individual counties at convincing accuracy during both waves in spring and fall of 2020. We demonstrate the dominating effect of local infection seeds and identify effective measures to attenuate the rapid spread. Our model has great potential to support decision makers on a state and community politics level to individually strategize their best way forward during the months to come.

**Supplementary Information:**

The online version contains supplementary material available at 10.1007/s10237-021-01520-x.

## Introduction

The year 2020 was dominated by the historic, global outbreak of the coronavirus disease, COVID-19 (SARS-CoV-2). With the first official cases being reported in December 2019 in Wuhan, China (Lu et al. 2020); cases have quickly spread over the entire world, culminating in the World Health Organization (WHO, 2020) declaring it a global pandemic on March 11, 2020.

Since then, each individual country had to find their own way to get the rapid spreading under control, to ‘flatten the curve,’ and to avoid a breakdown of the healthcare system. Different strategies of shutdown as well as travel and contact restrictions have been implemented in different countries and states, with more or less evidence for their success (Chinazzi et al. 2020; Maier and Brockmann 2020; Fang et al. (2020). While most countries managed to get the first wave of rising infections in spring under control, loosened restrictions in many European countries over the summer were followed by another steep increase and the widely feared (Xu and Li 2020), yet well predicted (Cacciapaglia et al. 2020) second wave in fall and winter. Other countries, such as the USA or Brazil, seemed to have moved from the first to the second wave more directly. This could be explained by the mere size of these countries, where a first wave may still propagate through more distant areas, while the second wave would already emerge (Cacciapaglia et al. 2020; Reiner et al. 2021).

Especially during the earlier phase of the pandemic, Germany had been given special attention. Firstly, its reported death counts were significantly lower than in neighboring countries such as Italy, Spain, or France (Johns Hopkins University 2020). This gave rise to the question whether Germany could serve as an important example for successful strategies to mitigate the impact now and during future pandemics. Secondly, its federal structure has led to different responses across its states to reduce human contact and prevent further spreading. While locally tailored reactions may benefit the people’s acceptance in the short run, widespread confusion over ever-changing rules and weary discussions to present a more united front against the virus may simultaneously undermine the effectiveness of the countermeasures, as seen specifically during the second wave. Thirdly, the Robert Koch Institute (RKI) provides locally resolved data on current cases in each county, enabling us to fit and test distributive models (Robert Koch Institute 2020a) that can predict the temporal and spatial outbreak dynamics.

Besides the medicinal effort to understand the disease, numerous mathematical studies have focused on modeling the outbreak dynamics of COVID-19, predict its future course, and provide scientific reasoning for political decisions. Typically, those epidemiology models follow the basic idea of compartmentalizing the entire population into different subgroups and modeling their coupled evolution with a set of ordinary differential equations (ODEs). Despite their inferiority in modeling the disease process based directly on data, which is possible with more complex memory-type models (Keimer and Pflug 2020; Kergaßner et al. 2020), their simplicity has paved their success. The most basic of such models is the SIR model, with groups of susceptible, infectious, and recovered or removed people, dating back to the 1920s (Kermack and McKendrick 1927). Overall, the course of a COVID-19 infection within such compartment models is quite well established by now. A susceptible is first exposed to the virus to become infected, before becoming infectious himself after some latency period. From here on, the infection may take various courses (An der Heiden and Buchholz 2020), ranging from no or mild symptoms for arguably the largest group of patients, to strong symptoms and patients who require hospitalization or even intensive care, before they recover or die from the disease. Severity mostly seems to depend on existing pre-conditions and general health, but also other reasons that have not yet been fully understood (Zhou et al. 2020; Yuan et al. 2020). The well-known SIR model has been extensively analyzed (Hethcote 2000) and extended to finer compartments (see Pastor-Satorras et al. 2015) for an earlier overview) that mimic the described course. Examples include the SEIR model with an exposed group, the SEIRD model to separate truly recovered and dead, an S(E)IQR model (Pedersen and Meneghini 2020; Hethcote et al. 2002; Jumpen et al. 2009) that puts known infections into a quarantined group that does not infect others, or the MSEIR model (Hethcote 2000) to include children with mother immunity, thus covering non-constant population sizes. Overall, these models have been abundantly applied to locally analyze COVID-19 outbreak dynamics in various countries, largely focusing on China (Kucharski et al. 2020; Maier and Brockmann 2020), Italy (Pedersen and Meneghini 2020), and the USA (Peirlinck et al. 2020).

However, models to predict the temporal and spatial spreading of the virus have so far been rather limited, while agent-based models (Eubank et al. 2004) successfully cover the high-resolution end at the level of individual people and their movement, especially the intermediate to high resolution on a state or county level is understudied territory—even though this is exactly where many of the political decisions are being made. Recent works analyze statistical relationships between neighboring provinces in China (Kang et al. 2020) or city districts in New York City (Cordes and Castro 2020). A variant of the SIRS model has previously been coupled to a reaction–diffusion model (Yamazaki and Wang 2017) to mathematically study cholera dynamics with partial differential equations (PDE). Colizza et al. have focused on the importance of the air travel network as a basis for global diffusion at a pandemic outbreak (Colizza et al. 2006). Following this strategy, Ellen Kuhl’s group at Stanford have coupled an air travel network to the SEIR model to understand spatial spreading in China, the USA (Peirlinck et al. 2020), and across Europe (Linka et al. 2020). Air travel is likely a major player at the very beginning of a global outbreak, in area-wise very large countries, such as the USA, and when lifting travel bans again (Linka et al. 2020). However, other factors may be more important in understanding the spatial distribution, for example across Europe, and within individual countries such as Germany. Zheng et al. similarly find higher correlations between case numbers and daily bus and train route frequencies, compared to air travel in China (Zheng et al. 2020). Especially with air travel being tightly controlled, operating at much lower volume, and dominating infection seeds present in all countries, we are in need of short- to mid-range network models on the county and province level (Prasse et al. 2020). At this level, models may be further informed by other, more societal factors such as income or occupation (Mollalo et al. 2020) While the global epidemic and mobility (GLEAM) model (Balcan et al. 2009) also includes air travel as the major source of wide-range disease spreading, it simultaneously models more localized commuting patterns that correspond well to traffic data in Germany, among other countries. The model is capable of explaining a large part of COVID-19 spreading in mainland China (Chinazzi et al. 2020).

As suggested by multiple previous studies (Ma et al. 2020; Bai et al. 2020), mildly or asymptomatic carriers account for the major share of new infections, and the large number of hidden infections facilitated global spreading (Li et al. 2020). Thus, here we model the spatiotemporal outbreak dynamics of COVID-19 in Germany with an SIQRD model that specifically distinguishes between the group of hidden infectious I and a Q group that holds people with known, quarantined infections that, consequently, do not infect others anymore (Kergaßner et al. 2020). Since Germany was several weeks behind China and Italy during the first phase of the pandemic with several global travel restrictions already in place, we couple the SIQRD model to the GLEAM mobility network to model short-range and intra-country interactions essential to locally resolve the evolution of the COVID-19 pandemic. We fit our model to both the first and second wave in Germany and compare differences and similarities in disease dynamics and political reactions, thereby demonstrating its robust predictive capabilities.

## Methods

We model the spatiotemporal outbreak dynamics of COVID-19 in Germany with an SIQRD model, coupled to a network model that allows for spatially distributed cross-county infections. We start out with the description of our basic compartment model that mainly governs the spread of the disease over time, and then continue with its spatial interactions and resolution. All simulations were implemented and performed in *Octave* 5.2.0 using packages *optim* 1.6.0, *statistics* 1.4.1, *io* 2.4.13, *parallel* 4.0.0, and *splines* 1.3.3.

### Basic SIQRD model

**Fig. 1:**
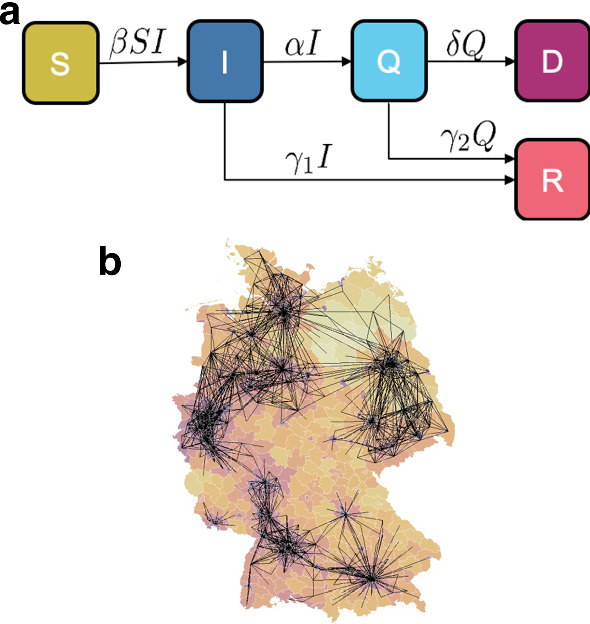
The basic and spatial SIQRD model. **a** Schematic of the basic SIQRD dynamics. **b** Major connectors (lines) between counties of the network mobility model to predict cross-county infections in the spatial SIQRD model. County color varies from yellow to dark purple with population density

In contrast to many existing studies that use a standard SEIR(D) model including a latency period between being infected and becoming infectious (Peirlinck et al. 2020; Linka et al. 2020), we focus on the difference between asymptomatic or mildly symptomatic, unknown cases that account for the major share of new infections (Ma et al. 2020; Bai et al. 2020), and people with noticeable symptoms. We integrate this knowledge and use an SIQRD model (Hethcote 2000; Jumpen et al. 2009) that specifically distinguishes between the infectious group I, representing a measure for the estimated total number of infections, and a group Q representing known and therefore quarantined infections, who do not infect others anymore (Pedersen and Meneghini 2020). In our case, the transition rate $\alpha$ from I to Q describes how long it takes for an infected person to be tested/detected and put in quarantine. The remaining three groups are considered as usual, where S represents the initial state of being susceptible, R represents truly recovered, and D dead. Some fraction of I can directly recover at rate $\gamma _1$ without ever being tested, overall representing the hidden infections that are never detected, while the remainder transitions to Q at rate $\alpha$. The rate $\gamma _2$ describes the rate to recover from a tested infection, while $\delta$ represents the rate to die from a confirmed infection (see schematic in Fig. 1). Thus, our model is based on the assumption that all those dying of COVID-19 are also identified through testing. Overall, normalized by population, we obtain the set of equations

1 $$\begin{aligned} {\dot{s}}&= -\beta s i \end{aligned}$$

2 $$\begin{aligned} {\dot{i}}&= +\beta s i {}&{} -\alpha i {}&{} - \gamma _1 i\end{aligned}$$

3 $$\begin{aligned} {\dot{q}}&=&+\alpha i {}&{} {}&{}- \gamma _2 q {}&{} - \delta q \end{aligned}$$

4 $$\begin{aligned} {\dot{r}}&=&+ \gamma _1 i&+ \gamma _2 q \end{aligned}$$

5 $$\begin{aligned} {\dot{d}}&=& + \delta q. \end{aligned}$$

Since we are neglecting disease-unrelated births and deaths, the total number of people *N* is constant, in Germany $N\approx 8e7$, such that $N \cdot [s + i + q + r + d] = S + I + Q + R + D = N$.

For easier interpretation of the model parameters, we introduce the dark ratio $\omega$ and the true mortality $\mu$(Kergaßner et al. 2020) as

6 $$\begin{aligned} \omega&= 1 + \frac{\gamma _1}{\alpha }, \end{aligned}$$

7 $$\begin{aligned} \mu&= \frac{\delta }{\gamma _2 + \delta } \frac{\alpha }{\alpha + \gamma _1}. \end{aligned}$$

Thus, we can replace the previous parameters $\alpha$ and $\delta$ by

8 $$\begin{aligned} \alpha&= \frac{\gamma _1}{\omega - 1}, \end{aligned}$$

9 $$\begin{aligned} \delta&= \gamma _2 \frac{\mu \omega }{1 - \mu \omega }. \end{aligned}$$

If we consider the stationary point $\hat{\{\bullet \}}$ when the pandemic has passed, we can directly relate the number of confirmed or tested infections to the estimated overall number of infections by ${\hat{I}} = \omega {\hat{Q}}$, while $\mu$ represents the fraction of all infected people that died. The true mortality $\mu$ is also often referred to as infection fatality rate (IFR). Further, the mortality can also be represented by the ratio of deaths over cumulative total infections, $\mu = {\hat{D}} / {\hat{I}}$. Since $\omega = {\hat{I}} / {\hat{Q}}$, we identify the stationary relationship

10 $$\begin{aligned} \frac{{\hat{D}}}{{\hat{Q}}} = \mu \omega . \end{aligned}$$

In other words, the measurable ratio *D*/*Q*, usually referred to as the case fatality rate (CFR), will eventually approach the number $\mu \omega$, demonstrating the inherent dependence of the two parameters, making it impossible to identify them separately from one another. During the course of the pandemic, however, CFR will not be constant (Dudel et al. 2020).

#### Modeling political measures and contact restrictions

Following our previous findings (Kergaßner et al. 2020), we introduce federal state-wise initial infection rates $\beta _{j0}$ with $j\in \{1,\ldots ,16\}$, and up to *m* reduction factors $\beta ^\text {red}_{m}$ representing imposed major restrictions. The reduction factors are assumed constant for all of Germany, but we respect their (potentially state-dependent) starting dates $T_{ji}$, which yields a piece-wise constant function for each state ${\hat{\beta }}_{ji}(t)$, such that the effective contact rate results in

11 $$\begin{aligned} \beta _j(t) = \beta _{j0} \prod _{i=1}^m {\hat{\beta }}_{ji}(t), \; \text {where} \; {\hat{\beta }}_{ji}(t) = {\left\{ \begin{array}{ll} 1, &{} \, \text {if} \; t < T_{ji}, \\ \beta ^{\text {red}}_{i} &{} \, \text {otherwise}. \end{array}\right. } \end{aligned}$$

Note that we consider the spring and fall infection waves separately, starting over with a new timeline and initial infection rate $\beta _{j0}$. In our fitting and prediction periods, we do not consider the time when restrictions are removed, but those can easily be included by resetting the corresponding reduction factor back to 1.0. For the first wave, we follow the implemented policies and consider two reduction factors $\beta ^{\text {red}}_{1}$ and $\beta ^{\text {red}}_{2}$ that represent the major restrictions of $i=1$ cancelling large events and $i=2$ general contact restrictions, together with school closings. Those two major restrictions were enacted simultaneously in all of Germany on March 8 and March 22, 2020, respectively. For the second wave in the fall, we consider only one reduction factor when the partial lockdown was enacted on November 2, 2020, in all of Germany.

#### Reproduction number

The model allows for straight-forward estimates on the initial and effective reproduction numbers $R_0$ and $R_{\text {eff}}$, respectively, which are well known in public and the general media as the number of infections originating from one infected person. Since this number is represented by the ratio between the influx and outflux of the hidden infectious group I in our model, we obtain the time-dependent upper-bound expression

12 $$\begin{aligned} R_{\text {eff}}(t) = \frac{\beta (t) s(t) \left[ \omega - 1\right] }{\omega \gamma _1} \le \frac{\beta (t)}{\gamma _1} \left[ 1 - \frac{1}{\omega }\right] = R_0(t), \end{aligned}$$

for $\omega > 1$. In relative numbers, $s=1$ during early stages of the pandemic. Note that model parameters such as $\alpha$ and $\omega$ may also vary over time, and a continuous or even randomized representation of the evolution of $R_{0}(t)$ (Linka et al. 2020) may potentially better explain the imperfect data. For better readability, we drop the time dependence of $\beta$ and $R_{0}$ in the following.

### Spatially resolved SIQRD model

In order to study the spatial dynamics of the spreading disease, we consider a network model on the resolution level of individual counties that allows for cross-county infections. We slightly adapt the GLEAM short- and mid-range mobility network (Balcan et al. 2009, 2010) and represent time-dependent cross-county infections via

13 $$\begin{aligned} \begin{aligned} B_{kl}&\!=\! {\left\{ \begin{array}{ll} \!\beta _k &{}\text {if k=l,} \\ \!c_k\! \sqrt{\!\beta _k \beta _l} \frac{N_k^\kappa N_l^\lambda }{N_{\text {max}}^{\kappa + \lambda }} \exp \left( \! - \frac{r_{kl}}{r}\!\right) &{}\text {else,} \end{array}\right. } \end{aligned} \end{aligned}$$

where $\beta _k$ are time-dependent infection rates and $c_k$ are the cross-county infection weights. We consider $N_{k}$ as the number of inhabitants in the largest city of county *k*, $N_{max}=3e6$ corresponds to the number of inhabitants in Germany’s largest city Berlin, and $r_{kl}$ is the distance between counties *k* and *l*. The exponential cross-county infection term is adopted from the Global Epidemic And Mobility (GLEAM) model, where the expression represents commuter flows between communities *k*, *l*. It can be tuned by three parameters $\kappa$, $\lambda$, and *r* that were fit to large amounts of commuting data to globally emulate their patterns, as described in Balcan et al. (2009) (Suppl. Table S1).

We note that $\beta _k$ and $c_k$ are identical for all counties within one federal state, thus introducing 16 additional model parameters $c_j$ with $j\in \{1,\ldots ,16\}$ which need to be calibrated based on reported data. Major connectors in this network over Germany are displayed in Fig. 1b (Kergaßner et al. 2020).

We introduce ${\overline{\bullet }}$ to denote the transformation of a vector $\bullet$ into a quadratic diagonal matrix, where the entries along the diagonal equal those of the vector. Then, for all counties $k=1,\ldots ,n_{\text {c}}$ joined in vector notation we obtain the set of normalized and reparameterized coupled SIQRD network differential equations

14 $$\begin{aligned} {\dot{{\mathbf {s}}}}&= - {\overline{{\mathbf {s}}}} ~ {\mathbf {B}}{\mathbf {i}}\end{aligned}$$

15 $$\begin{aligned} {\dot{{\mathbf {i}}}}&= + {\overline{{\mathbf {s}}}} ~ {\mathbf {B}}{\mathbf {i}}- \frac{\gamma _1}{\omega - 1} {\mathbf {i}}- \gamma _1 {\mathbf {i}}\end{aligned}$$

16 $$\begin{aligned} {\dot{{\mathbf {q}}}}&= + \frac{\gamma _1}{\omega - 1} {\mathbf {i}}- \gamma _2 {\mathbf {q}}- \gamma _2 \frac{\mu \omega }{1 - \mu \omega } {\mathbf {q}}\end{aligned}$$

17 $$\begin{aligned} {\dot{{\mathbf {r}}}}&= + \gamma _1 {\mathbf {i}}+ \gamma _2 {\mathbf {q}}\end{aligned}$$

18 $$\begin{aligned} {\dot{{\mathbf {d}}}}&= + \gamma _2 \frac{\mu \omega }{1 - \mu \omega } {\mathbf {q}}, \end{aligned}$$

### Initial conditions and parameter fitting

We use data from the Robert Koch Institute (RKI) that is available for each county in Germany over time (Robert Koch Institute 2020a). Since RKI infection data have limited information content, we had to fix several parameters from other data describing the course of infection. Following the works of An der Heiden and Buchholz (2020), we set the mortality to $\mu =0.006$. As described by various other works (An der Heiden and Buchholz 2020; Zhou et al. 2020; Dorigatti et al. 2020), the time to recover from a confirmed infection varies between 18 and 25 days, while milder, often undetected infections last for about 5 to 10 days. Based on these data, we chose $\gamma _2 = 0.04$ and $\gamma _1 = 0.067$, directly following from the assumption that about 50% of cases are asymptomatic (National Institute of Infectious Diseases Japan 2020) and infectious, yet undetected, over a time span of 7.5 days. Following the assumed mortality and an average time-to-death for a confirmed infection of 15 days (An der Heiden and Buchholz 2020), state-wise dark ratios can be directly read out from the RKI-reported death toll (Robert Koch Institute 2020a), averaged over the last week of the respective fit. We emphasize that the calculated dark ratio is a direct consequence of the fixed mortality of $\mu =0.006$ and is not a result related to our modeling framework or the fitting procedure. If the mortality was chosen differently due to new findings as the pandemic continues, the dark ratios would change accordingly. We decided against fitting the D group over time, due to the disparate mortality across the age structure of infected people (Dudel et al. 2020), which is not very well represented in SIR-type, rate-based models (Kergaßner et al. 2020). The age-dependent mortality was clearly visible in Germany, especially during the early stages when younger people were over-proportionally affected, with correspondingly low death rates.

For our parameter optimization, we solve the nonlinear set of ordinary differential equations (ODEs) from the start date onward in time using an ODE45 integration scheme with variable time-stepping and evaluate the daily new and cumulative infection numbers via spline interpolation.

To identify the free model parameters for our spatially resolved county model, we followed an identical cascade optimization strategy for both wave scenarios. Using state-wise identified dark ratios $\omega _j$ and constant $\mu$, $\gamma _1$, and $\gamma _2$, we first used a 16-node network model connecting each federal state to obtain a Germany-wide average $\beta$ and reduction factors $\beta ^{\text {red}}_1$ (and $\beta ^{\text {red}}_2$ for the first wave) by fitting the cumulative data for Germany. We then considered state-wise data to fit $\beta _{j0}, j\in \{1,\ldots ,16\}$, while keeping $c=1$. As initial values, we set the number of confirmed infections on our start dates as the size of $Q_0$. To obtain an appropriate size of $I_0$, we estimated the change rate of *Q* on our start date via an exponential function and then exploit $I_0 = {\dot{Q}}_0 [\omega - 1] / \gamma _1$.

For the first and second waves, respectively, we fitted the cumulative number of confirmed infections from the RKI for the time periods from March 3 until April 22 and October 2 until November 21 with the cumulative entries in our *Q* group, normalized by the maximum number of RKI infections. We also considered the cumulative deceased on the last day of the fitting period, for which the death count was reliable. For the first wave, data delay was not an issue, and we were able to take deceased numbers on April 22, identical to the last day of the fitting period for cumulative infected. For the second wave, a significant number of deaths were still being reported over a period of two to three weeks after the release of the used data set, as analyzed in detail in Sect. 3.1. Therefore, we integrated the cumulative deceased numbers during the second wave two weeks earlier on November 7. In addition, we included the change rate of infections over the respective last week of the fit in April and November into the residual vector. Note that cumulative infections at time *T* reported by the RKI correspond to the integrated influx $\tilde{{\mathbf {Q}}}(T)$ into the *Q* group of our SIQRD model, such that the fitted expression is obtained via

19 $$\begin{aligned} \tilde{{\mathbf {Q}}}(T) = \int _{t=0}^{T} \frac{\gamma _1}{\omega -1 } {\mathbf {I}}(t) dt, \end{aligned}$$

where individual entries *j* of the vector ${\mathbf {I}}(t)$ can be determined at state level, i.e., $I_j(t) = N_j i_j(t), j=1,\ldots ,16$, or also evaluated at county level. Finally, we increased the resolution to full county level, amounting to a network of 401 nodes. We used a gradient descent algorithm to iteratively fit state-wise cross-county weights $c_{j}, j\in \{1,\ldots ,16\}$ to re-balance the changed influence of the larger network, while keeping the previously determined state-wise $\beta _{j0}$ fixed.

An important component of spatially resolved predictions is the choice of appropriate initial conditions. Besides the mere size of $I_0$, the initial infectious population, we also needed to specify its spatial distribution at the start date of the county-wise simulation. For the first wave, this distribution had to reflect the fast dynamics, lacking experience of the general public, and a presumably high dark ratio. Due to several days delay between the outbreak in different federal states and, naturally, their different population size, we therefore selected the RKI-reported distribution on March 17 and scaled its magnitude down to obtain the overall number as determined on March 3, state-wise amounting to $\langle I_{0,i} \rangle = 3075$ (see Methods, Suppl. Table S2). The ratio between Q and I was computed as before. For the second wave, the situation is much simpler, as basically all counties were affected by the viral spread, such that data alone provided consistent initial conditions.

The entire fitting procedure, except for obtaining the final cross-county weights $c_{j}$, was done using a particle swarm optimization (PSO) scheme. PSO is a meta-heuristic inspired by the behavior of natural animal swarms. It uniformly initializes a swarm of particles in a multidimensional search space, such that the objective function is evaluated at the current position of each particle. Particles communicate their best position amongst each other. Thereby, individual particle direction and speed are updated depending on their own and the overall best position in search space found up to this point. This way, the swarm broadly covers the bounded search space (Helwig 2010) and likely converges to a global optimum, while exploring many local minima along the way (Schmitt and Wanka 2015). The scheme balances broad coverage with fast convergence and provides valuable information on explored samples.

### Statistical analysis

To validate the model, we evaluated the temporal and spatial correlation between model predictions and RKI data by computing the Pearson correlation coefficient $r_P$, the coefficient of determination $R^2 = r_P^2$ and the corresponding *p* value to assess statistical significance via the function [$r_P$,*p*] = *corrcoef*(...) in *Octave* 5.2.0. We further compared the daily averaged root-mean-squared distance (RMSD) of detected infections (compartment Q) between RKI data ${Q}^{\text {RKI}}_j$ and model values, following Eq. (19), during the fitting and prediction intervals for each federal state $j\in \{1,\ldots ,16\}$ via

20 $$\begin{aligned} \text {RMSD}_j = \sqrt{\frac{1}{N_jT}\sum _{t=1}^{T}\left[ {Q}^{\text {RKI}}_j(t)-{\tilde{Q}}_j(t)\right] ^2}, \end{aligned}$$

and analogously for the deceased (compartment D).

## Results

### Quality of data

**Fig. 2:**
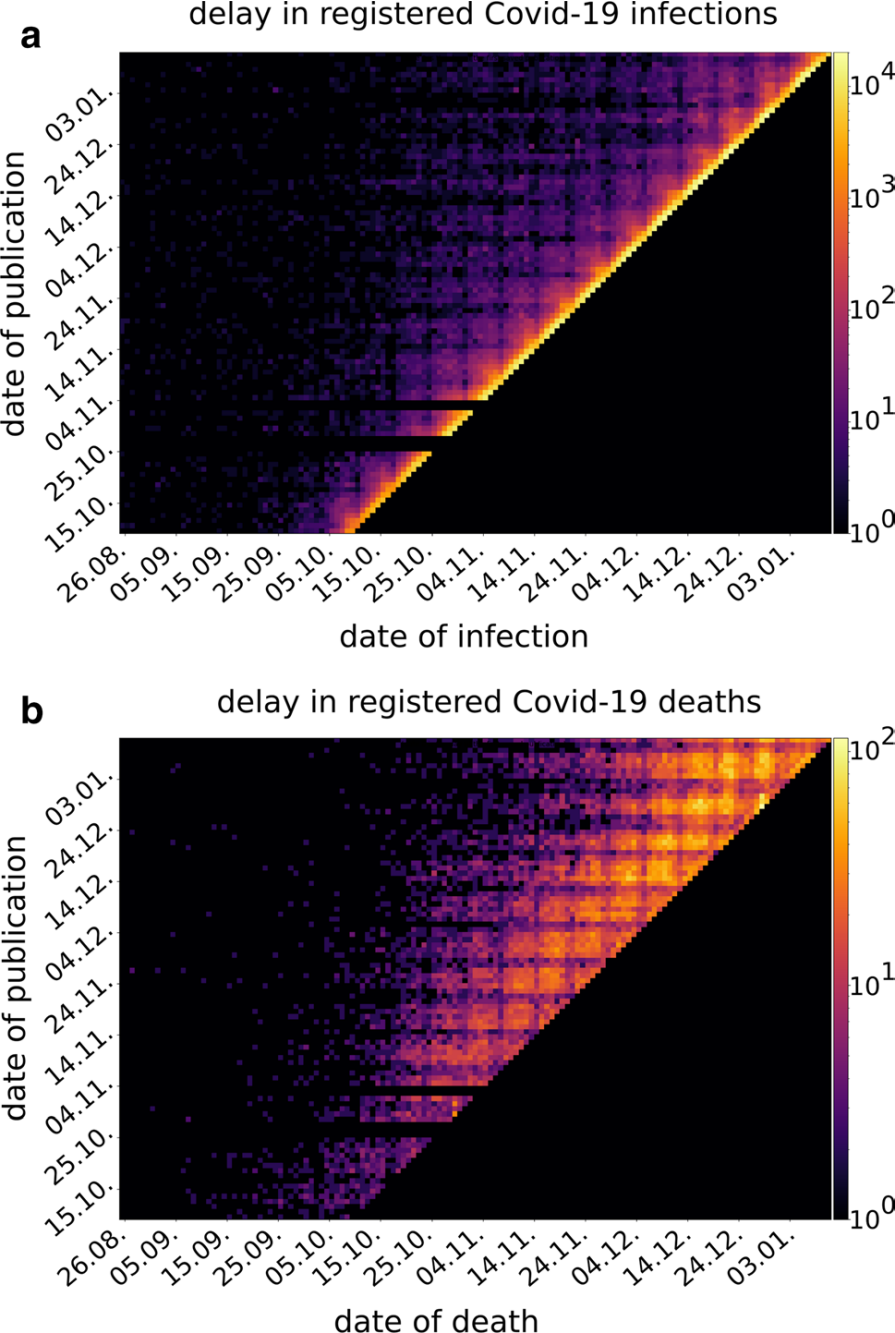
Data delay. Data delay in the number of registered infections (*Q*, (**a**)) and deaths (*D*, (**b**)) according to color scale, comparing the actual date of infection/death (horizontal axis) and the date the infection/death is published (vertical axis). Lines are empty where data was not available

To obtain dependable results during fitting, we first analyzed the quality of data available in Germany and its information content to identify our model parameters. To this end, Fig. 2 shows the time delay in reported infection cases and deaths, i.e., the days between an infection (a death) is known and it being reported during the period of the second wave in fall. A very similar situation is observed during the first phase of the pandemic (not shown). It can be inferred from the graph that only after about three to five days delay, a relatively complete picture of current infection numbers is available. Note that this time delay does not account for the incubation period between becoming infected and becoming infectious, but merely the time of information flow. For deaths, the situation is even worse. Here, we have to wait for one to three weeks before the numbers become sufficiently stable. The weekly pattern of daily new infections/deaths is clearly visible, represented by the grid-like appearance of Fig. 2. Overall, this delay is important to consider when fitting model parameters directly from data. We therefore used a data set from November 27 for our fitting period of March 3 to April 22 and October 2 to November 21. To avoid any impact from reporting delay on the fitting results, cumulative deceased were only considered on November 7 when fitting the second wave.

**Fig. 3:**
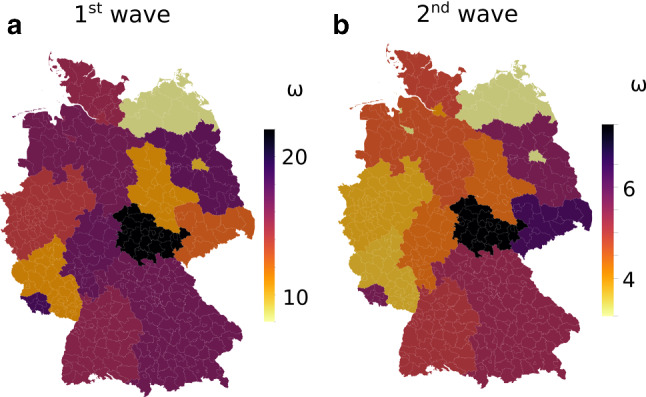
Federal state-wise dark ratios. State-wise estimated dark ratios following from a Germany-wide constant mortality during the first (**a**) and second (**b**) wave

As mentioned in Sect. 2.1, dark ratio $\omega$ and mortality $\mu$ are inherently coupled and cannot be identified exclusively from reported infection numbers and deceased. However, by assuming a Germany-wide identical mortality of $\mu =0.006$ based on medical data (An der Heiden and Buchholz 2020), we obtain estimates on state-wise dark ratios $\omega _j$ (Fig. 3) by fitting to the individually reported death tolls, with $\langle \omega _j\rangle \approx 14.84$ and $\langle \omega _j\rangle \approx 4.67$ during the first and second wave, respectively (see Suppl. Tables S2 and S3). Note that the mortality is at the lower end of reported values in the literature, thereby providing an upper bound on the dark ratios. Interestingly, we observe a low, negative correlation (Pearson coefficient $r_P = -0.3998$, $R^2=0.1599$ $p<0.1249$) between state-wise $\omega _j$ and performed per-capita tests, which varied significantly from about $0.02\%$ of the population in Saarland to about $1.43\%$ in Berlin during the first wave (test numbers from April 24, Suppl. Table S2 (Robert Koch Institute 2020b)). The much higher test capacities, nearly tripling from 0.8 million per week in calendar week 16 to 2.1 million per week in week 49, 2020 (Robert Koch Institute 2020c), very well match and thus explain the significantly lower dark ratios during the wave in fall, despite their correlation being less prominent at higher testing rates. Here, we observe stronger correlations of the dark ratios with reported case fatality rates, which is to be expected according to Eq. 10.

### Optimizing the spatially resolved SIQRD model

**Fig. 4:**
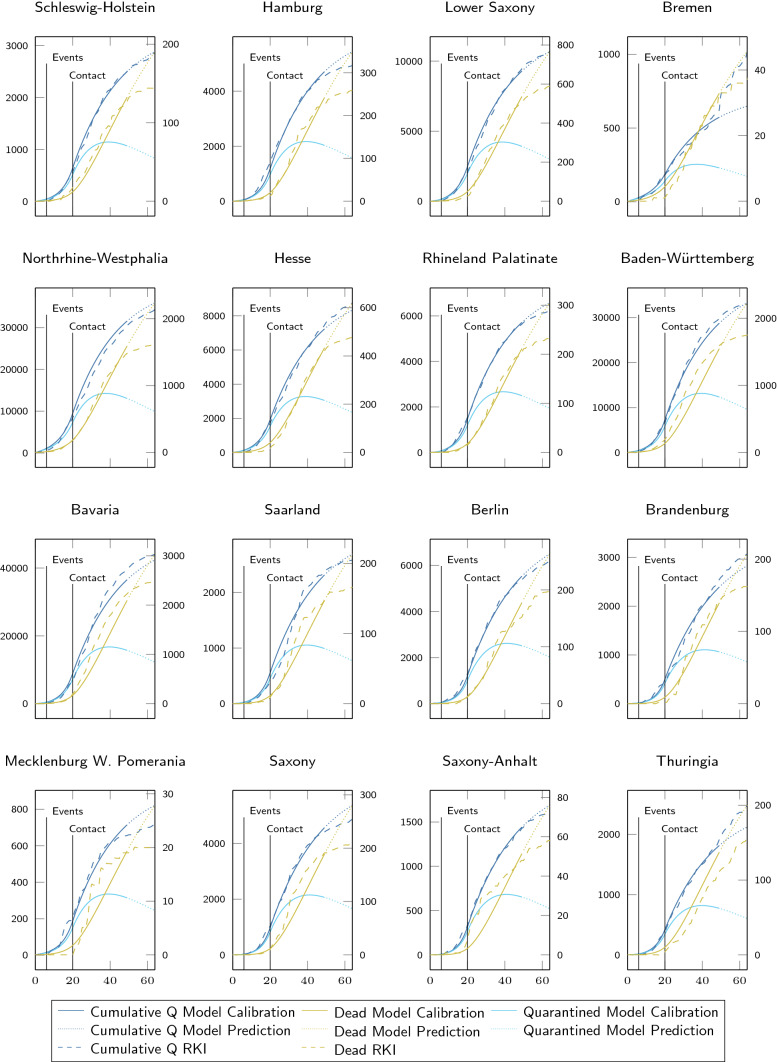
State-wise calibrations and predictions of the spatially resolved SIQRD model for the first wave. The evolution of cumulative quarantined infections *Q* (left axis, blue; current *Q* in teal) and dead count on the last day (right axis, ocher) during the first wave were state-wise fitted to RKI data (dashed) from March 3 to April 22 (*x* in days since March 3). The change from solid to dotted lines highlights the end of the calibration and start of the prediction phase for the infected and the only value fitted for the deceased. Thin vertical lines denote changes in $\beta$

**Fig. 5:**
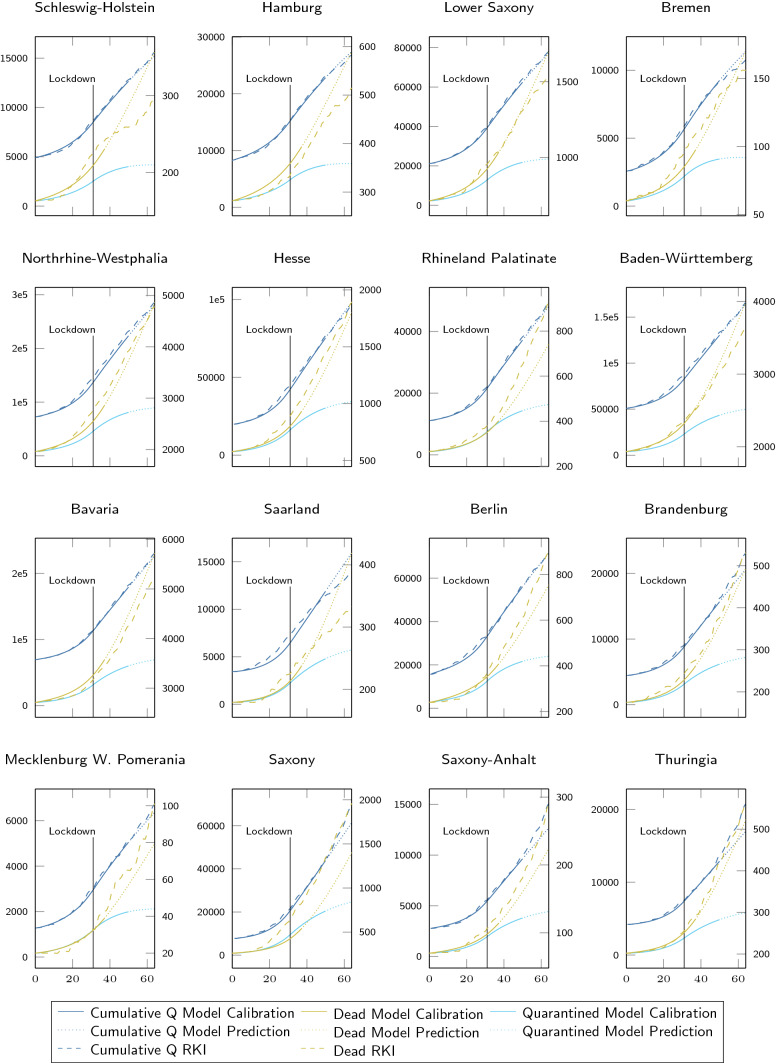
State-wise calibrations and predictions of the spatially resolved SIQRD model for the second wave. The evolution of cumulative quarantined infections Q (left axis, blue; current Q in teal) and dead count on the last day (right axis, ocher) during the second wave were state-wise fitted to RKI data (dashed) from October 2 to November 21 (*x* in days since October 2). The change from solid to dotted lines highlights the end of the calibration and start of the prediction phase for the infected and the only value fitted for the deceased. The thin vertical line denotes the change in $\beta$

Figures 4 and 5 demonstrate that the optimized spatially resolved SIQRD model with 401 network nodes representing each county of Germany well reproduces the cumulative confirmed cases in each of its federal states from March 3 until April 22 as well as October 2 until November 21. Importantly, they demonstrate how the model further extends two-week predictions that well match the evolution of data for both waves. For cumulative infection data reported by the RKI (Robert Koch Institute 2020a), we find good agreement on the temporal evolution for both waves (see Suppl. Tables S4 and S5 for correlation measures). The model slightly overestimates the number of deaths during the end of the first wave of the pandemic. However, considering that besides a delay in death counts, one might expect also a certain number of undetected deaths related to COVID-19, relativizing the deviations. When comparing both waves, it becomes obvious that the severe contact restrictions during the first wave successfully slowed down the viral spread, while the (partial) lockdown during the second wave did not produce similar reductions in daily new infections.

**Fig. 6:**
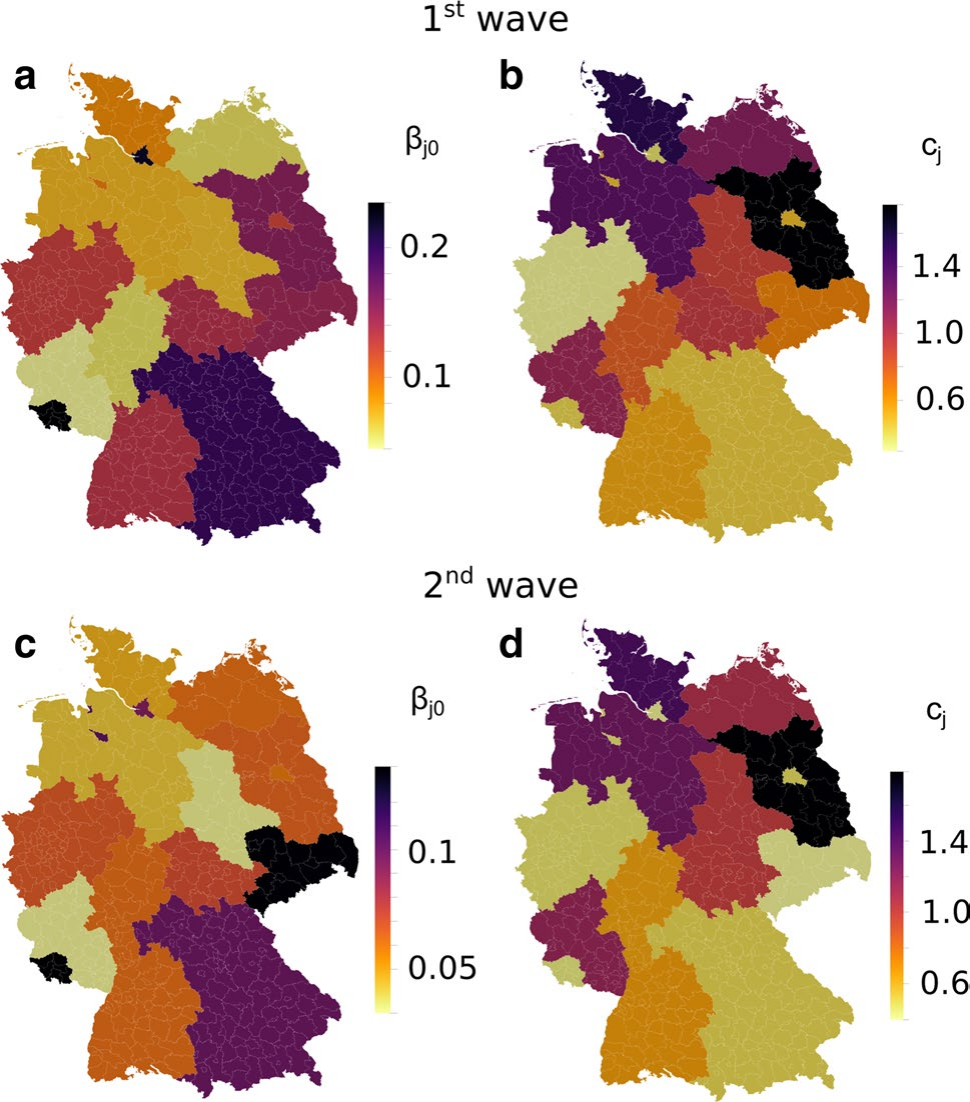
Optimized state-wise model parameters. Illustration of state-wise optimized values for $\beta _{j0}$ (**a, c**) and $c_{j}$ (**b, d**) for $j\in \{1,\ldots ,16\}$ for the first (**a, b**) and second (**c, d**) wave

Figure 6 displays the corresponding federal state-wise $\beta _{j0}$ and $c_{j}$. Importantly, our preliminary investigations had shown that it is not sufficient to provide a single $\beta$ valid in entire Germany, even with state-wise dark ratios $\omega _j$. It is, therefore, key to calibrate infection rates $\beta _{j0}$ differing between federal states. The reduction factors, however, are modeled identical for all of Germany, merely considering their state-wise starting date. They are optimized to $\beta _{\text {red},1}=0.84$ and $\beta _{\text {red},2}=0.14$ for the first wave, and $\beta _{\text {red},1}=0.53$ for the second wave. We note that the optimized parameter values are to a certain extent affected by the choice of the disease-specific input parameters, i.e., the mortality $\mu$ as well as the recovery parameters $\gamma _1$ and $\gamma _2$, which were fixed using data on the course of disease. However, the fact that the model provides good estimates of cumulative cases for the next two weeks and the qualitative parameter trends are not affected.

On average, the intra-county infection rates $\beta _{j0}$ are about half as large as for the basic SIQRD models (Pedersen and Meneghini 2020), leading us to believe that about half of infections occur through cross-county interactions. We observe an opposite trend between $\beta _{j0}$ and $c_{j}$. This can in part be attributed to their inherent parameter dependence, as demonstrated by an extended sensitivity analysis of the model (Suppl. Table S6, for details on the analysis see Kleuter 2007). Nevertheless, due to our cascading optimization scheme, $\beta _{j0}$ and $c_{j}$ are never optimized simultaneously. Since we scale the calibrated network mobility model (Balcan et al. 2009) by $c_{j}$, a trade-off between usual inter-state contact and the spread of the disease is suggested. For example, northern (touristy) regions obtain higher inter-state contact rates, suggesting that they observed relevant inflow from other states, compensating for their relatively sparse intra-state cross-county network. The distributions show very similar patterns for both the first and second wave.

**Fig. 7:**
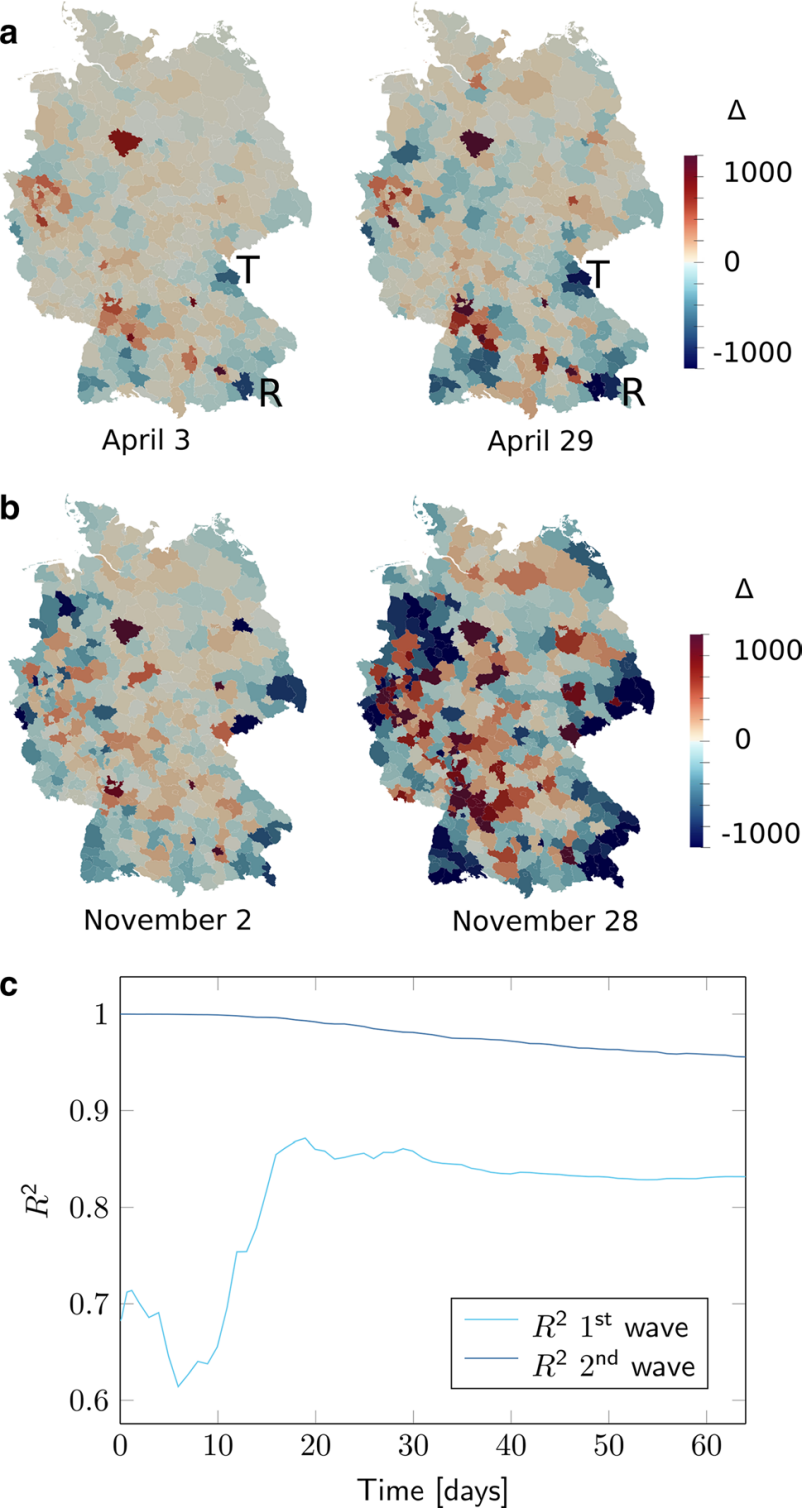
County-level validation. County-level evaluation of cumulative quarantined infections Q on two randomly selected dates during the first (**a**) and second (**b**) wave, showing the difference of infections SIQRD−RKI, increasing from blue to red, with indicated counties Tirschenreuth (T) and Rosenheim (R). For comparability and better contrast, we fixed the color scale to [−1000,1000] from [−717,2166] on April 3, [−1280,4402] on April 29, [−1518,4001] on November 2, and [−3056, 12449] on November 28. (**c**) Temporal evolution of the spatial Pearson coefficient of determination across all counties for both waves. Time is measured in days since the start of the corresponding wave, March 3 and October 2, respectively

### County-wise predictions

We then analyzed how well the federal state-wise fitted model represented the infections on a county level. Figure 7a and b shows the difference of cumulative entries in Q between our model predictions and RKI-reported numbers on two dates in April and November, respectively, without further county-wise fitting or optimization. During both waves, we find a strong, significant correlation of the spatial distribution over time, as displayed in Fig. 7c, demonstrating an overall high level of accuracy of our mesoscale model. The initially larger discrepancies during the first wave originate from the difficulty of obtaining suitable initial conditions when only few counties were affected. However, the model representation on county level improves after two to three weeks. Larger differences that catch the eye occur in Hanover, a large, well-populated county that seems to be over-represented in the model, and a few southern, more rural counties that suffered from more infections than predicted by our model. The most prominent examples are the indicated counties Rosenheim (R) and Tirschenreuth (T). Per-capita infections in the hot spot city Mitterteich in Tirschenreuth temporally surpassed the numbers in New York City (Johns Hopkins University 2020), and one of the most stringent curfews was put in place to contain the virus spread (Landratsamt Tirschenreuth: 2020).

During the second wave, both infection numbers and absolute differences between model and data are on a higher level. Strikingly, most counties where the model seems to underestimate outbreak dynamics are on borders to neighboring countries (Fig. 7b). This is not surprising: While borders were closed during the first wave, they remained open during fall, allowing for more exchange between Germany and its neighbors, which is not included in the model. Nevertheless, our county-wise distribution matches very well, as indicated by the high correlations in Fig. 7c.

**Fig. 8:**
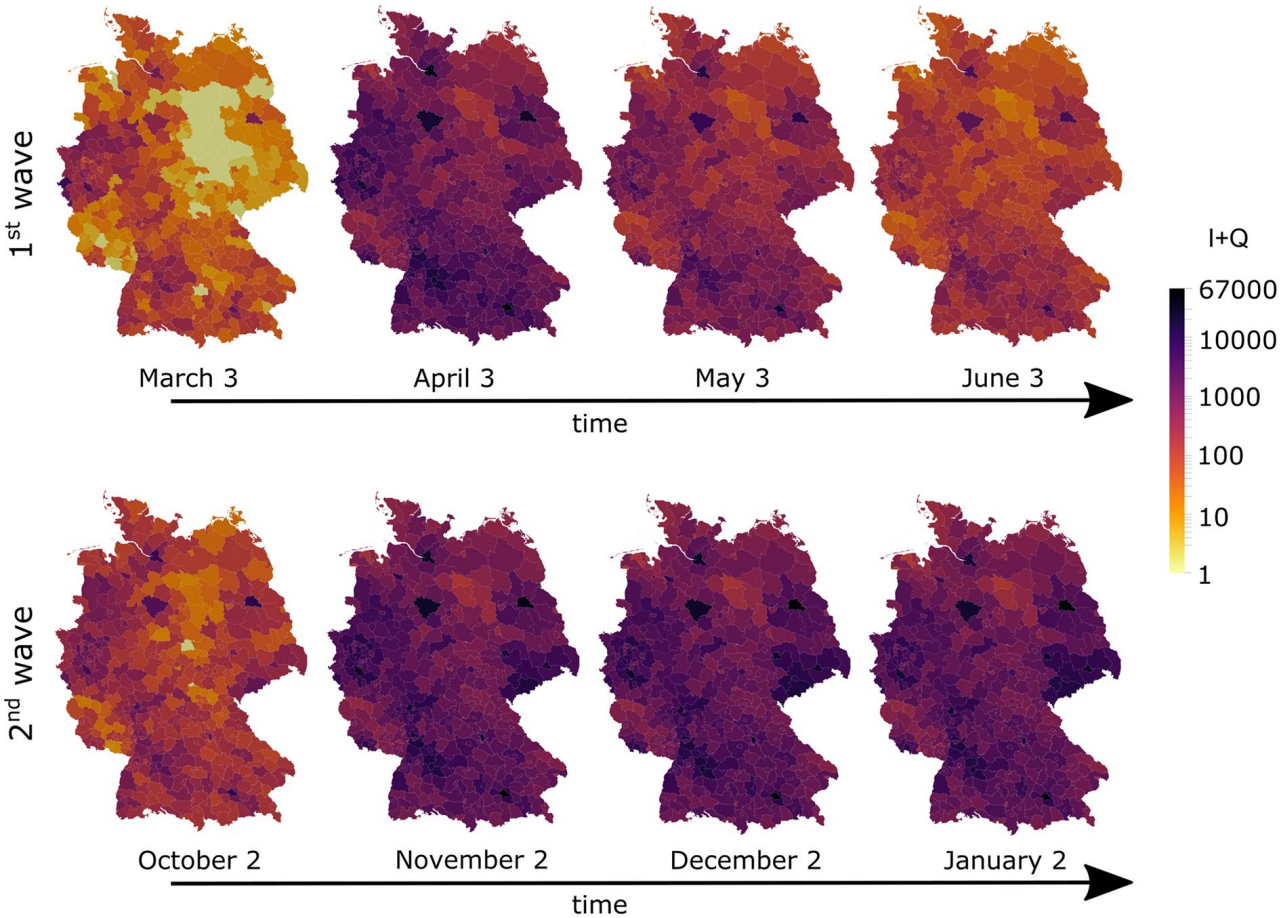
Spatiotemporal model predictions. Spatiotemporal snapshots of the epidemic spread (overall number of active infections *I* + *Q*) across Germany during the first (top) and second (bottom) wave, continuing with the identified reduced infection rates $\beta _j$, respectively

Following this validation, we used our model to obtain a complete spatiotemporal timeline of the COVID-19 spreading. Figure 8 and supplementary movies S1 and S2 show the predicted spatial distribution of all infections, i.e., combined entries of I *and* Q, evolving March to June and October to January, respectively, at the resolution of individual counties, assuming the particular contact reduction factors stay in place. The snapshots nicely capture the fading first wave, while infection levels stay high during the second wave.

### The effect of seeds

The spreading of COVID-19 in Germany and the initiation of the first wave had allegedly evolved from two major hubs: (1) a carnival event in the county Heinsberg (H) in the Ruhr area in Western Germany, and (2) returnees from skiing holidays in Northern Italy and Austria, with a large share tracing back to Ischgl (I). With the aid of our spatially resolved model, we investigated how these sources may have affected the spreading throughout Germany.

In Heinsberg, we set 10% of the population in the I group, i.e., $I_0=4195$, corresponding to about 65% of the population found infected in Streeck et al. (2020). In Ischgl, we started out with an I group of 30 times the number of its inhabitants, amounting to $I_0 = 1617 \cdot 30 = 48510$, to represent the major tourist flow through the town and returnees from other ski resorts in Austria and Italy. We further chose parameters *c* and $\beta _{j0}$ equal to the highest ones found in a German state to initiate the spreading, corresponding to the value in Brandenburg and Saarland, respectively.

**Fig. 9:**
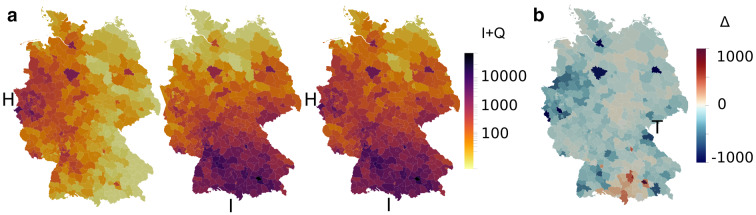
Seed effects. (**a**) Scenario comparison on April 03 close to the epidemic peak, with seeds spreading from Heinsberg (H, left), Ischgl (I, center), and both cities (H+I, right). (**b**) Difference plot of cumulative infections Q between our simulation with seeds from Ischgl and Heinsberg vs RKI data on April 03. Color scale cropped from [-3317,3645] to [-1000,1000] for better contrast

Figure 9a shows the distributions of confirmed, quarantined infections Q resulting from initial outbreaks in Heinsberg (left) and Ischgl (center) only, as well as the distribution resulting from the combination of both (right), demonstrating the balance of local and far-reached infections with our model. Red hot spots appear in nearly all major urban areas across Germany, but rural spreading occurs much more in areas closer to the seeds. When evaluating differences of infections from their combined spreading to RKI data (Fig. 9b) near the German peak on April 03, we find that, despite increased differences very close to the Southern borders, the overall state-wise distribution in BY is nearly identical in quality ($R^2$ from 0.8642 to 0.8420). This demonstrates the dominating influence of returnees from skiing holidays in Italy and Austria represented by the Ischgl seed for the Germany-wide initiation of the spread. Tirschenreuth (T), however, seems to have suffered from their very own, less documented super-spreading event. On the other hand, the distribution in NW differs more to the data ($R^2$ from 0.9009 to 0.5964). We observe several underrepresented counties, suggesting that cases from Heinsberg alone spread less, similar to the localized situation in Tirschenreuth. The overall similarity between Figs. 9a and 8, as well as Figs. 9b and 7a, is in line with previous epidemiological findings (Streeck et al. 2020; Felbermayr et al. 2020) that the virus indeed spread from the Southern and Western states of Germany, with Ischgl and Heinsberg as two major representative seeds.

## Discussion

We have shown that a spatially resolved SIQRD model can well explain and predict the temporal and spatial outbreak dynamics of COVID-19 in Germany during both waves encountered so far. The reparameterized model specifically includes undetected, hidden infections as a separate compartment, revealing a direct coupling between mortality, testing efforts, and the dark ratio. Our systematic refinement from Germany-wide to spatially resolved county-level predictions has revealed that we require different values for dark ratios $\omega _j$, infection rates $\beta _{j0},$ and cross-county weights $c_{j}$ in each federal state of Germany to accurately capture the spreading of COVID-19. At first, this is quite surprising considering that various other studies with single-node, country-wide models have predicted single infection rates $\beta$ that are quite similar for different countries, such as Germany, France, or Spain (Yuan et al. 2020; Pedersen and Meneghini 2020). However, this can be attributed to their low spatial resolution and high infection numbers, which average out any spatiotemporal fluctuations. Higher-resolution information as presented here thus comes at the cost of more complex model requirements.

Differences in $\omega _j$ can in part be attributed to variable testing activities. It is important to note, however, that the varying dark ratio alone is not enough to account for the different outbreak dynamics in the federal states of Germany. Rather, there seems to be a non-negligible influence of habits and mentality that drive different infection rates $\beta _{j0}$, together with random factors and local super-spreader events such as the carnival celebrations in Heinsberg (Streeck et al. 2020). We observe an opposite trend between $\beta _{j0}$ and $c_{j}$ (Fig. 6), suggesting that some states (mostly Northern and distant from initial seeds) received more infections from neighboring states, while states close to epidemic seeds suffered more from localized infections. Generally, the adapted mobility network tended to overestimate cross-county terms in densely populated areas, where the pandemic seemed to have a larger reduction effect on typically observed traffic patterns, manifested by smaller weights $c_{i}$.

It has become clear from our analysis that the data we currently have at our disposal makes it impossible to provide ‘true’ parameter sets that uniquely describe the evolution of the pandemic. However, despite the deduced interdependence of mortality $\mu$ and dark ratio $\omega$, the relationship to testing activities holds regardless, underlining the importance of broad, fast testing. Interestingly, the test capacity increased from 0.8 to 2.1 million per week from the first to the second wave (Robert Koch Institute 2020c). This corresponds well to the identified decrease in the dark ratio from an average of 14.8 (Table S2) to an average of 4.7 (Table S3). Increased (antibody) testing can help strengthen our confidence bounds on $\omega$ and $\mu$ in the future. Importantly, our analysis showed that data sets must be taken with caution, as newly reported cases still affect past infection and death numbers for days and even weeks before becoming robust. This must be considered during the fitting procedure.

Our spatially resolved model can predict the temporal evolution of infections on a county level fairly well. It captures the fact that the probability for new incoming infections and higher spreading is generally larger in densely populated urban environments. However, we have also seen a few rather rural counties with high infection numbers that were much less hit in our predictions, e.g., the county Tirschenreuth in Eastern Bavaria (Fig. 7a). We postulate that such locally over-proportionate case counts can be attributed to rather random super-spreading events, which may pop up anytime and can easily be included in our model, but are hard to predict in advance.

Exploiting our county-level resolution, we were able to infer the effect of infections stemming from selected seeds, such as two major hubs for Germany, Heinsberg and returning travellers from Ischgl in Austria. Our model demonstrates how the outbreak dynamics in Germany were initially driven by these two major seeds and spread from there throughout the rest of the country (Fig. 9). Nevertheless, from our difference analysis we found that Heinsberg itself was significantly more contained than Ischgl. Taken together, these observations corroborate that refraining from traveling and large events are two key interventions that can effectively attenuate the spreading of infectious diseases such as COVID-19. In addition to reducing travel (Linka et al. 2020), or mobility in general (Linka et al. 2020), our model results support the notion that containing local seeds is a further important aspect to get the viral spread under control.

The county-wise comparison between both waves impressively showed how our model, being limited to Germany, underestimated infections in counties close to the border. While severely limited travel during the first wave successfully reduced new infections, political actions during the second wave were much less successful. While it is important to balance the economic and health consequences during this pandemic, it is clear that policies must focus on mobility and contact reduction to get the spread under control again.

The presented model has certain limitations that we aim to address in the future. One drawback of all SIR-type modeling approaches is that they hardly account for the various courses of disease: In such rate-dependent models, some appear as infinitely long infectious. To prevent this issue from significantly affecting our optimized parameters, we only considered the latest dead count in our fitting procedure (see Methods). Still, we plan to adapt our model to integrate detailed information on specific courses of disease within a memory-based or delayed ODE, as introduced, for example, in Keimer and Pflug (2020 and Kergaßner et al. (2020).

While our county-level models well captured the spatiotemporal outbreak dynamics and could even be extended to city-level resolution (Kergaßner et al. 2020), general SIR-type models then seem to approach their validity limit, while stochastic effects start to become more important. Whereas SIR-type compartment models may capture the spread on a macro- and mesoscale level, at very low infection numbers or high spatial resolution, individual agent-based models (Eubank et al. 2004; German et al. 2020) are required to accurately predict the course of the epidemic. It is noteworthy, though, that current agent-based models may scale up to $\approx 50.000$ agents, leaving quite a gap to mesoscale models like ours. We will investigate how coupling both types of methods in a multi-scale model can close this gap in the future. Similarly, explicitly integrating uncertainty via stochastic models (Palomo et al. 2020) may help to further improve model predictions at high spatial resolution and low to medium infection numbers, potentially providing insights into optimal strategies for political action.

Overall, our refined predictions could provide a trustworthy rationale to elaborate community-wise reopening and closing strategies, and inform on distribution strategies of vaccination and/or antibody tests once available for the general public. The optimized model can be directly adopted to estimate the effects of loosened restrictions, potential new seeds, new waves, or other influencing factors on the resolution of individual counties when continuously fitted to new incoming data. It can thus be a valuable tool to support (political) decision makers to appropriately react to future developments of the COVID-19 situation and expediently avoid a third wave.

## Supplementary Information

Below is the link to the electronic supplementary material.

Supplementary file1 (PDF 142 kb)

Supplementary file1 (MP4 4110 kb)

Supplementary file1 (MP4 2858 kb)

## Data Availability

Data on the spread of COVID-19 in Germany are publicly available from the respective resources as cited in the paper. Simulation scripts are available on GitHub: https://github.com/chburkhardt/SIQRD_spatial.
