# Supplementary material for: Spatiotemporal modeling of first and second wave outbreak dynamics of COVID-19 in Germany
Source: Biomech Model Mechanobiol. 2021 Oct 6;21(1):119–33. doi: 10.1007/s10237-021-01520-x (PMC8493548; doi:10.1007/s10237-021-01520-x)
Supplement: Supplementary file 1 — Supplementary file1 (PDF 142 kb) [file 10237_2021_1520_MOESM1_ESM.pdf]

Supplementary Information

Supplementary tables

r_{kl}[km]	κ	λ	r[km]
≤ 300	0.46	0.64	82
> 300	0.35	0.37	1

Table S1: Parameters for the network mobility model as derived in [2].

state	β	c	ω	I_0	tested [%]	tested positive [%]
Schleswig-Holstein (SH)	0.09	1.54	15.45	877	0.27	3.98
Hamburg (HH)	0.22	0.37	15.06	853	0.23	8.96
Lower Saxony (NI)	0.07	1.36	16.64	1898	0.49	4.78
Bremen (HB)	0.10	0.48	15.96	726	0.15	1.93
Northrhine-Westphalia (NW)	0.13	0.29	14.33	22973	0.83	8.14
Hesse (HE)	0.06	0.80	17.25	2565	0.42	10.42
Rhineland Palatinate (RP)	0.04	1.11	10.95	362	0.87	7.34
Baden-Württemberg (BW)	0.14	0.55	15.72	9648	0.42	11.92
Bavaria (BY)	0.20	0.44	16.78	7470	0.70	10.36
Saarland (SL)	0.23	0.41	18.57	426	0.02	5.26
Berlin (BE)	0.12	0.48	9.82	642	1.43	5.95
Brandenburg (BB)	0.16	1.77	17.49	200	0.40	5.01
Mecklenburg W. Pomerania (MV)	0.06	1.20	8.05	171	0.27	2.28
Saxony (SN)	0.15	0.67	12.59	141	0.54	6.03
Saxony-Anhalt (ST)	0.07	0.92	10.85	0	0.53	3.20
Thuringia (TH)	0.14	0.97	21.95	254	0.53	3.47
mean	0.12	0.83	14.84	3075	0.51	6.19
STD	0.06	0.44	3.49	5801	0.33	2.96

Table S2: Optimized state-wise parameters for the spatially resolved SIQRD model, percentage of population tested and proportion of positive tests by April 24, 2020.

state	β	c	ω	I_0	tested [%]	tested positive [%]
SH	0.05	1.45	4.89	2473	5.03	1.71
HH	0.10	0.40	4.08	4429	2.20	2.19
NI	0.04	1.32	4.70	10191	4.87	2.90
HB	0.11	0.46	3.25	1187	0.59	2.25
NW	0.07	0.46	3.82	28668	10.26	3.96
HE	0.06	0.65	4.41	9537	3.85	6.38
RP	0.03	1.18	3.68	3598	4.59	4.27
BW	0.06	0.67	5.11	19005	4.63	3.24
BY	0.10	0.50	5.45	25098	9.05	2.65
SL	0.13	0.44	5.74	1063	0.77	2.26
BE	0.06	0.48	2.49	6286	4.55	3.76
BB	0.06	1.79	5.71	1923	2.97	3.50
MV	0.06	1.10	3.17	497	2.56	1.93
SN	0.13	0.40	6.35	5349	7.48	6.78
ST	0.03	1.02	4.43	1370	2.75	1.73
TH	0.07	1.01	7.37	1761	2.53	4.11
mean	0.07	0.83	4.67	7652	4.29	3.35
STD	0.03	0.42	1.23	8621	2.63	1.47

Table S3: Optimized state-wise parameters for the spatially resolved SIQRD model, percentage of population tested and proportion of positive tests by November 17, 2020.

state	R^2 Fit Q	RMSD Fit Q	R^2 Pred. Q	RMSD Pred. Q	R^2 Fit D	RMSD Fit D	R^2 Pred. D	RMSD Pred. D
SH	0.9976	1.84e-05	0.9480	1.29e-05	0.9882	2.23e-06	0.9242	9.37e-06
HH	0.9962	6.64e-05	0.9746	1.85e-04	0.9853	6.27e-06	0.9429	3.08e-05
NI	0.9969	2.83e-05	0.9907	1.58e-05	0.9880	2.81e-06	0.9640	1.39e-05
HB	0.9840	4.19e-05	0.9717	3.62e-04	0.9867	2.52e-06	0.8098	7.87e-06
NW	0.9967	7.94e-05	0.9930	7.73e-05	0.9913	2.85e-06	0.9929	2.24e-05
HE	0.9959	3.19e-05	0.9851	5.67e-05	0.9948	2.05e-06	0.9849	1.36e-05
RP	0.9988	1.78e-05	0.9891	5.45e-05	0.9936	1.71e-06	0.9357	1.06e-05
BW	0.9984	7.05e-05	0.9826	6.70e-05	0.9738	1.51e-05	0.9786	2.43e-05
BY	0.9932	1.47e-04	0.9897	1.91e-04	0.9824	1.65e-05	0.9774	2.23e-05
SL	0.9786	1.47e-04	0.9767	5.45e-05	0.9759	1.28e-05	0.9189	2.78e-05
BE	0.9981	2.81e-05	0.9933	6.33e-05	0.9766	2.52e-06	0.8828	1.14e-05
BB	0.9900	3.62e-05	0.9822	8.50e-05	0.9857	3.24e-06	0.9504	9.77e-06
MV	0.9897	1.73e-05	0.9774	5.44e-05	0.9375	1.57e-06	0.3923	2.89e-06
SN	0.9973	2.27e-05	0.9852	9.16e-05	0.9674	4.79e-06	0.9585	1.03e-05
ST	0.9987	9.93e-06	0.9876	1.97e-05	0.8991	3.21e-06	0.8989	5.11e-06
TH	0.9972	1.84e-05	0.9813	1.18e-04	0.9947	6.29e-06	0.9738	1.67e-05

Table S4: State wise R^2 values and per-capita RMSD for the fit of cumulative infection numbers and deaths from March 03 to April 22, 2020 as well as for their model predictions from April 23 to May 06, 2020.

state	R^2 Fit Q	RMSD Fit Q	R^2 Pred. Q	RMSD Pred. Q	R^2 Fit D	RMSD Fit D	R^2 Pred. D	RMSD Pred. D
SH	0.9982	5.47e-05	0.9890	7.99e-05	0.9258	3.52e-06	0.9673	1.73e-05
HH	0.9989	1.31e-04	0.9805	2.76e-04	0.9938	9.83e-06	0.9545	3.21e-05
NI	0.9993	1.03e-04	0.9922	1.25e-04	0.9910	2.24e-06	0.9852	1.27e-05
HB	0.9972	2.28e-04	0.9919	6.71e-04	0.9805	9.97e-06	0.9731	1.08e-05
NW	0.9976	3.61e-04	0.9959	3.22e-04	0.9910	8.11e-06	0.9953	6.03e-06
HE	0.9964	2.83e-04	0.9944	2.12e-04	0.9938	1.26e-05	0.9932	1.70e-05
RP	0.9988	1.39e-04	0.9941	2.31e-04	0.9972	9.48e-06	0.9966	3.68e-05
BW	0.9956	3.08e-04	0.9922	1.36e-04	0.9923	3.57e-06	0.9959	2.29e-05
BY	0.9994	1.24e-04	0.9965	2.48e-04	0.9969	8.77e-06	0.9914	2.80e-05
SL	0.9819	5.13e-04	0.9926	1.43e-03	0.9474	9.40e-06	0.9812	5.95e-05
BE	0.9957	2.92e-04	0.9906	1.36e-04	0.9935	7.68e-06	0.9894	3.03e-05
BB	0.9969	1.09e-04	0.9917	6.14e-04	0.9930	4.86e-06	0.9931	1.22e-05
MV	0.9980	4.64e-05	0.9883	1.28e-04	0.9885	2.94e-06	0.9840	8.30e-06
SN	0.9975	2.12e-04	0.9943	1.21e-03	0.9814	5.28e-05	0.9961	1.25e-04
ST	0.9978	7.10e-05	0.9904	6.36e-04	0.9942	4.40e-06	0.9813	1.91e-05
TH	0.9964	1.03e-04	0.9889	1.09e-03	0.9960	5.55e-06	0.9899	1.65e-05

Table S5: State wise R^2 values and per-capita RMSD for the fit of cumulative infection numbers and deaths from October 02 to November 21, 2020 as well as for their model predictions from November 22 to December 05, 2020.

Cross-correlation	Parameter 1	Parameter 2
1.000	β(MV)	c(MV)
1.000	β(SL)	c(SL)
0.999	β(HH)	c(HH)
0.998	β(SN)	c(SN)
0.997	β(BY)	c(BY)
0.997	β(ST)	c(ST)
0.997	β(HB)	c(HB)
0.997	β(RP)	c(RP)
0.997	β(BE)	c(BE)
0.996	β(BW)	c(BW)
0.995	β(BB)	c(BB)
0.992	β(NW)	c(NW)
0.992	β(SH)	c(SH)
0.989	β(NI)	c(NI)
0.989	β(HE)	c(HE)
-0.980	Major Events	γ_1
-0.970	ω	γ_1
0.955	ω	Major Events
-0.908	Contact Restrictions	γ_1
0.856	ω	Contact Restrictions
0.823	Major Events	Contact Restrictions
0.794	β(BE)	β(BB)
0.745	β(BB)	c(BE)
0.739	β(BE)	c(BB)
0.725	ω	β(NW)
0.723	Contact Restrictions	β(BY)
0.705	Contact Restrictions	c(BY)
0.685	c(BE)	c(BB)
-0.672	γ_1	β(BY)
0.669	ω	c(NW)
-0.664	γ_1	β(NW)
0.649	Major Events	β(BY)
0.648	ω	β(NI)
-0.641	γ_1	c(BY)
0.639	β(SH)	β(HH)
0.619	Major Events	β(NW)
0.613	Major Events	c(BY)
-0.609	γ_1	c(NW)
0.606	β(SH)	c(HH)
0.594	β(HH)	β(NI)
0.593	ω	c(NI)
0.593	Major Events	β(NI)
-0.589	γ_1	β(BW)
0.579	β(SH)	β(NI)
0.578	Contact Restrictions	β(BW)

Table S6: Analysis of the correlation of parameters according to [25], considering the residual from the second step of the optimization cascade for the first wave. An absolute value of 0 means no correlation, whereas values of ± 1 corresponds to a fully positively/negatively coupled pair of parameters.

Supplementary movies

Movie S1: Spatio-temporal prediction of Covid-19 outbreak dynamics in Germany at county level from March 3 until June 3.
Movie S2: Spatio-temporal prediction of Covid-19 outbreak dynamics in Germany at county level from October 2 until January 2.
